# Evolving therapeutic strategies in glioblastoma: traditional approaches and novel interventions

**DOI:** 10.1007/s13205-025-04493-1

**Published:** 2025-08-28

**Authors:** Chirag Shetty, Rajesh Tamatta, Namdev Dhas, Abhishek Kumar Singh

**Affiliations:** 1 Manipal Centre for Biotherapeutics Research, Manipal Academy of Higher Education, Manipal, India; 2 Department of Pharmaceutics, Manipal College of Pharmaceutical Sciences, Manipal Academy of Higher Education, Manipal, India

**Keywords:** Glioblastoma, Immunotherapy, CAR-T-cell therapy, Cancer vaccines, Nanotherapy

## Abstract

Glioblastoma (GBM) is the most aggressive primary brain tumor in adults and is characterized by rapid growth, diffuse infiltration, and resistance to conventional therapies. This review explores pathophysiology, molecular mechanisms, and therapeutic advancements of GBM. GBM is highly heterogeneous and can be classified into molecular subtypes based on genetic and epigenetic alterations, influencing patient prognosis and treatment response. Despite advances in surgical techniques, chemotherapy, and radiotherapy, survival remains limited, with a median of 15–18 months. Emerging therapeutic strategies, including immunotherapy, tumor treatment, oncolytic virotherapy, and nanotechnology-based drug delivery, are under investigation to increase treatment efficacy. Immunotherapy, particularly checkpoint inhibitors and CAR-T-cell therapy, has potential but faces challenges due to the immunosuppressive microenvironment of GBM. Oncolytic viruses and personalized vaccines aim to trigger antitumor immune responses, whereas nanotechnology-based approaches enhance drug delivery across the blood‒brain barrier (BBB). This review highlights the urgent need for multimodal strategies that integrate novel therapies with existing standards to improve patient outcomes. Future research should focus on overcoming treatment resistance, leveraging molecular profiling for personalized medicine, and exploring innovative drug delivery systems.

**Supplementary Information:**

The online version contains supplementary material available at 10.1007/s13205-025-04493-1.

## Introduction

Glioblastoma (GBM), also known as glioblastoma multiforme, is recognized as the most aggressive and lethal primary brain tumor in adults and is classified by the World Health Organization as a grade IV astrocytoma (Louis et al. 2007). This type of tumor is characterized by rapid growth, extensive infiltration, and notable resistance to treatment, accounting for 15% of primary brain tumors and more than 50% of gliomas (Ostrom et al. 2019). Its origin in pathogenesis is the glial cells of the central nervous system, specifically the astrocytes that are charged with providing structural and functional support to neurons (Louis et al. 2016).

Current GBM classification emphasizes molecular heterogeneity, with discrete subtypes identified based on specific genetic changes, gene expression patterns, and clinical behaviors (Verhaak et al. 2010). This was subsequently formalized by the World Health Organization (WHO) (Rehfuess et al. 2019). Current characterization involves a composite assessment of epigenetic, genetic, transcriptomic, and metabolic features such as age, sex, and the contribution of noncoding RNAs (Verdugo et al. 2022). An accepted classification system divides gene expression profiling and has revealed that GBM consists of four discrete molecular subtypes: classical, mesenchymal, proneural, and neural. This classification emphasizes the biological heterogeneity of GBM and applies to both prognostic assessment and the development of targeted therapeutic strategies (Steponaitis and Tamasauskas 2021). The classical subtype is generally associated with EGFR amplification (Talasila et al. 2013). The mesenchymal subtype is associated with NF1 alterations and increased microglia/macrophage infiltration, the proneural subtype is commonly associated with PDGFRA amplification, and a characteristic gene expression profile defines the neural subtype (Alentorn et al. 2012; Neftel et al. 2019). These subtypes exhibit varied clinical behaviors, responses to therapy, and prognoses, underscoring the importance of molecular classification in individualized treatment strategies.

Technological advancements in neuroimaging, molecular diagnosis, and treatment options have greatly enhanced GBM management. Evolved imaging modalities such as MRI with advanced sequences, diffusion tensor imaging (DTI), and perfusion imaging have improved early diagnosis and planning of surgery (Salama et al. 2017). Despite advancements, the overall prognosis for GBM patients is poor, with a median survival of 15–18 months following standard therapy regimens that involve maximal surgical resection, radiation therapy, and chemotherapy with temozolomide (TMZ) (Stupp et al. 2009).

GBM remains a major challenge, and novel therapies are needed. Current research focuses on immunotherapy that harnesses and enhances the body's immune system to recognize and eliminate tumor cells. It works by stimulating immune responses or by inhibiting regulatory pathways that limit immune activation (Lu et al. 2024), targeted treatments and personalized approaches. Understanding GBM biology and its tumor microenvironment is key to improving survival and patient quality of life.

## Pathophysiology and molecular mechanisms

**Fig. 1:**
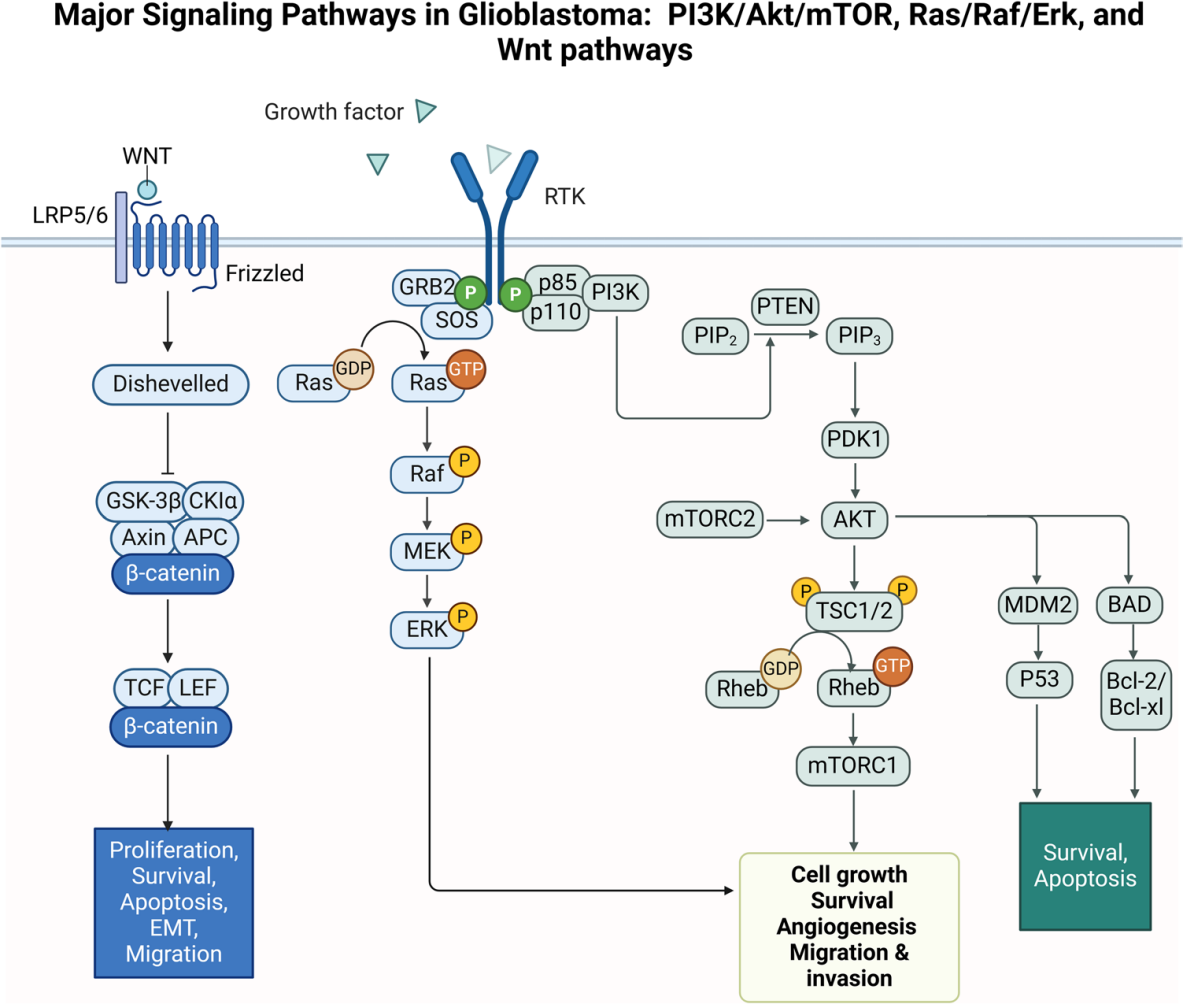
Major signaling pathways involved in GBM: A schematic illustrates three key pathways driving GBM progression. In the Wnt/β-catenin pathway, Wnt signaling inhibits the β-catenin destruction complex, leading to β-catenin accumulation and the activation of genes promoting proliferation, survival, and epithelial‒mesenchymal transition. The PI3K/Akt/mTOR pathway is activated by receptor tyrosine kinases (RTKs), which stimulate PI3K to convert PIP2 into PIP3 and activate Akt and mTOR to drive cell growth, angiogenesis, and metabolism, with PTEN acting as a negative regulator. In the Ras/Raf/Erk pathway, RTKs activate a Ras-driven kinase cascade (Raf-MEK-ERK) that enhances proliferation, migration, and survival. Crosstalk among these pathways strengthens GBM resistance to apoptosis and therapy

GBM results from a complex interplay among epigenetic changes, genetic alterations, and signaling pathways (Behrooz et al. 2022). Excessive proliferation, survival, migration, invasion, and angiogenesis are induced by aberrant activation of RTKs such as PDGFR, EGFR, and IGF-1R (Khabibov et al. 2022; Rai et al. 2024). The PI3K/Akt/mTOR and MAPK/ERK pathways are central to GBM progression, with PI3K/Akt/mTOR hyperactivation often due to PTEN loss or receptor amplification driving growth, metabolism, survival, and chemoresistance, whereas MAPK/ERK signaling promotes mitogenic transcription and cell cycle progression (Shahcheraghi et al. 2020; Khabibov et al. 2022). The intrinsic apoptotic pathway, governed by the Bcl-2 family and the tumor suppressor p53, is frequently disrupted by TP53 mutations, allowing tumor cells to evade cell death and further promoting resistance to therapy (Muller and Vousden 2013; Thumpati et al. 2025). A comprehensive understanding of these intertwined signaling networks is crucial for GBM growth, alongside the development of combination therapies to overcome resistance mechanisms and improve clinical outcomes (Fig. 1).

Epigenetic alterations, including MGMT promoter methylation, histone modification, and noncoding RNA modification, also control gene expression and treatment response (Sun et al. 2011; Sturm et al. 2012). A combination of epigenetic and genetic alterations provides a complex molecular platform for GBM pathogenesis.

## Glioblastoma therapy

Therapeutic interventions for alleviating GBM are described below.

### Standard treatments

#### Surgical interventions

Surgical resection is fundamental in GBM management, primarily to alleviate mass effects, reduce tumor burden, and obtain diagnostic tissue. Recent technological advances have improved surgical outcomes. For example, advanced imaging modalities such as diffusion tensor imaging, FET-PET, and MR spectroscopy aid in delineating tumor margins, although converting 2D imaging into the 3D surgical field remains challenging because of brain shifts (Georgiopoulos et al. 2017; Verburg et al. 2020).

While surgery is the foundation of GBM treatment, a balance between maximal safe resection and neurological preservation remains crucial for optimizing outcomes. The infiltrative nature of GBM makes complete surgical removal impossible, leading to inevitable recurrence.

#### Chemotherapy

Chemotherapy remains a key component of GBM treatment, complementing surgery and RT. Temozolomide (TMZ) is the standard first-line agent and is administered in the Stupp regimen concurrently with daily TMZ with external beam RT, followed by six cycles of adjuvant TMZ. Compared with RT alone, this protocol improves the 2-year survival rate to 27.2% (Stupp et al. 2009). Bevacizumab, an anti-VEGF antibody, is primarily used in recurrent GBM to increase progression-free survival and quality of life by reducing peritumoral edema, although its impact on overall survival in newly diagnosed patients is inconsistent (Friedman et al. 2023). Investigational combinations, such as TMZ with lomustine (CeTeG/NOA-09 trial), are being explored to overcome intrinsic chemoresistance (Weller et al. 2023).

Nitrosoureas such as lomustine and fotemustine are particularly relevant in the recurrent setting. Lomustine, which is administered at 80–110 mg/m^2^ every 6 weeks, has demonstrated clinical efficacy in recurrent GBM, with trials reporting median overall survival ranging from 8.6 to 9.8 months, particularly in patients with MGMT-methylated GBM (Wirsching et al. 2014; Weller et al. 2023). Chemotherapy is limited by resistance mechanisms, systemic toxicity, and the blood‒brain barrier, which restricts drug delivery. While TMZ remains the backbone of chemotherapy, novel regimens and drug delivery approaches are needed to increase therapeutic efficacy.

#### Radiotherapy

Radiotherapy is integral to GBM management, with innovations aimed at increasing tumor control while minimizing damage to healthy tissue. Advanced techniques such as intensity-modulated radiotherapy (IMRT), volumetric modulated arc therapy (VMAT), and hypofractionation allow for better dose conformity, especially when tumors are adjacent to critical structures (Burnet et al. 2014; Cheung et al. 2022). Proton beam therapy (PBT) exploits the Bragg peak to deliver high doses directly to the tumor, showing significant survival benefits compared with conventional RT, albeit with concerns regarding late radiation necrosis and limited accessibility (Matsuda et al. 2023). Brachytherapy and intraoperative radiotherapy (IORT) have shown promising reductions in local progression rates; for example, IORT has been shown to reduce local progression from typical rates of 80% to 35.5% (Phillips et al. 2012).

GBM is highly radioresistant, and recurrence often occurs within previously irradiated regions, complicating retreatment. RT remains critical in GBM therapy, but strategies to increase tumor radiosensitivity and minimize toxicity are essential for further improving outcomes.

#### Tumor treating fields (TTFs)

TTF offers a noninvasive treatment modality by delivering alternating electric fields that disrupt mitosis, leading to cell death. TTF is now included in the NCCN guidelines as a standard treatment for GBM based on prospective randomized trials that demonstrated improved overall survival, although its real-world adoption remains limited (Crompton et al. 2024). The Optune device, approved by the FDA in 2011 for recurrent GBM, applies low-intensity, intermediate-frequency fields via scalp electrodes (Stupp et al. 2017). Despite its clinical benefits, slow adoption is attributed to cost, daily usage requirements, and mixed guideline recommendations (Weller et al. 2021). TTF therapy requires long-term daily usage and is associated with compliance challenges, high costs, and limited real-world adoption. While TTF is a promising adjunct to GBM treatment, further studies are needed to refine its optimal use and integration into multimodal therapy.

Conventional treatment modalities, including chemotherapy and radiation, have broad effects, harming normal cells and causing significant side effects. By contrast, immunotherapy is more specific in that it leverages the immune system to target cancer cells specifically, with the added benefit of long-term immune memory.

### Immunotherapy

#### Immune checkpoint inhibition therapy

**Fig. 2:**
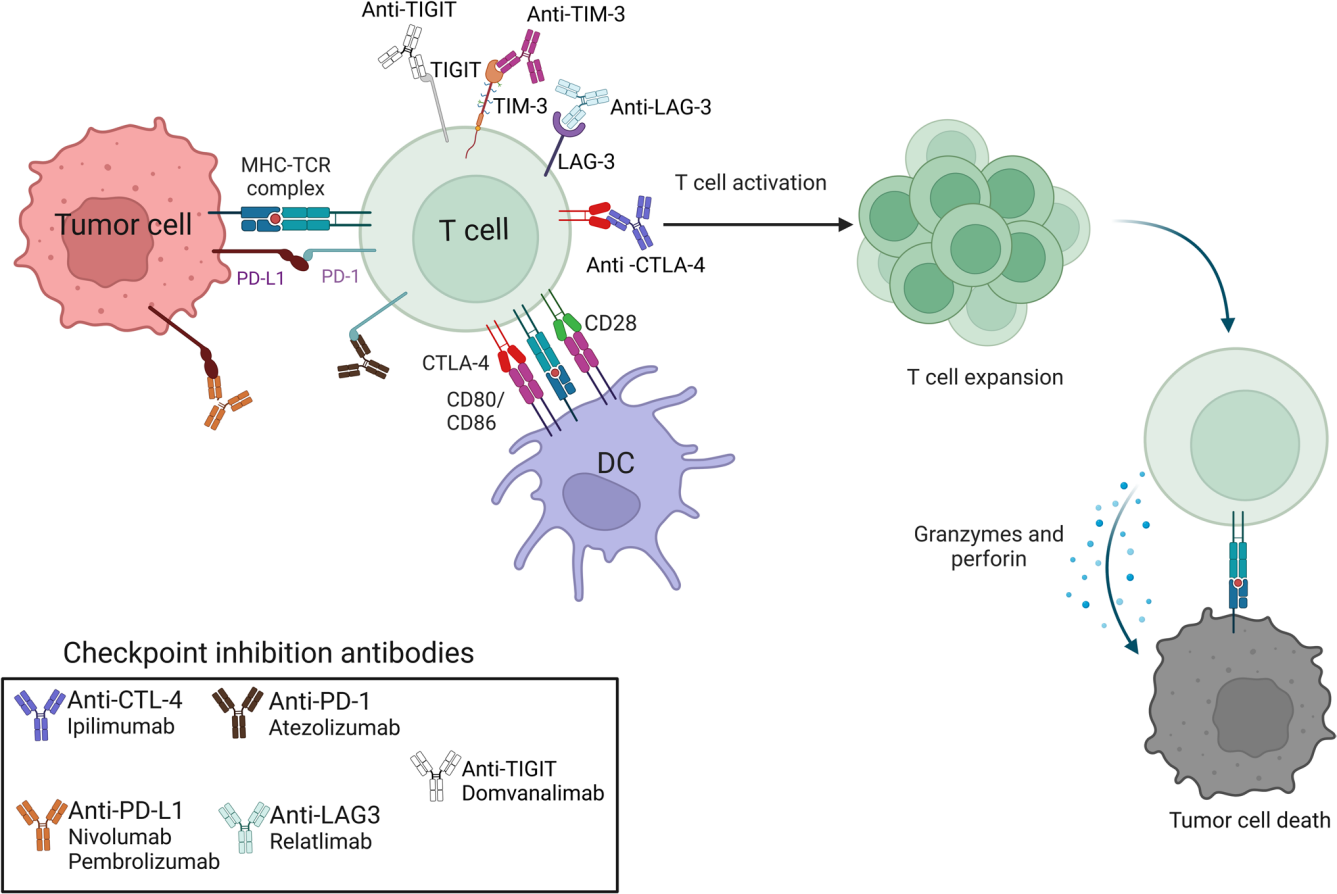
Checkpoint Inhibition in GBM: Schematic showing that checkpoint inhibition reactivates T-cellmediated cytotoxicity in GBM. Tumor antigens on MHC molecules activate T cells, but PD-L1 binding to PD-1 suppresses their function. Dendritic cells enhance activation via CD80/CD86-CD28 signaling, whereas inhibitory receptors (CTLA-4, TIM-3, LAG-3, and TIGIT) further impair responses. Monoclonal antibodies (e.g., ipilimumab, nivolumab, and pembrolizumab) block these inhibitory signals, restoring T-cell activity, clonal expansion, and cytotoxic molecule release (granzyme and perforin) to induce tumor cell death

ICIs have revolutionized cancer treatment; they aim to enhance the body's immune response against tumor cells by blocking inhibitory pathways to promote antitumor activity, yet their application in GBM remains challenging. Unlike traditional therapies such as chemotherapy and radiation, which target both healthy and cancerous cells, immunotherapy offers enhanced specificity by modulating immune responses to selectively attack tumor cells. Key advantages include reduced systemic toxicity, improved safety profiles, and the potential to establish long-term immune memory, which helps prevent recurrence (Liu et al. 2024) (Fig. 2).

Numerous clinical trials of ICIs in adult GBM patients have shown immunologic changes (Table S1), such as increased immune cell infiltration and elevated chemokine expression, but these changes have not been consistently translated into meaningful clinical benefits (Schalper et al. 2019). A major obstacle is achieving sufficient drug delivery across the BBB, suggesting that localized administration may be more promising than systemic approaches.

Neoadjuvant approaches have also been explored. In a phase II trial (NCT02550249), neoadjuvant nivolumab altered the immune microenvironment in recurrent, resectable GBM, although clear postsurgical benefits were limited, with only a few patients experiencing prolonged survival (Schalper et al. 2019). Similarly, a phase II trial of neoadjuvant pembrolizumab (NCT02337686) demonstrated minimal T-cell infiltration and a predominance of CD68⁺ macrophages (de Groot et al. 2020).

Ongoing studies are investigating novel ICI combinations and alternative approaches. The PERGOLA trial (NCT03899857) is evaluating pembrolizumab combined with TMZ-based radiochemotherapy in newly diagnosed GBM, whereas a randomized phase II study (NCT02794883) is comparing tremelimumab plus durvalumab to monotherapies, assessing endpoints such as progression-free survival and overall survival. Emerging therapies targeting costimulatory receptors are under investigation; for example, a phase I trial (NCT02658981) evaluating anti-LAG-3 or urelumab (anti-CD137) alone and in combination with anti-PD-1 therapy revealed prolonged survival in a subset of patients (Lim et al. 2020). Additionally, trials targeting CD27 in combination with nivolumab (NCT02335918) and a small, randomized phase II trial of neoadjuvant pembrolizumab (Cloughesy et al. 2019) have demonstrated enhanced antitumor immune responses, although these studies are limited by small sample sizes. Resistance mechanisms, such as PTEN mutations that drive immunosuppressive profiles, further contribute to the limited efficacy of PD-1 blockade in GBM (Zhao et al. 2019).

Novel combinatorial strategies are also under evaluation. A phase 0/I trial (NCT04656535) is testing the safety and immunologic effects of combining domvanalimab (AB154) and zimberelimab (AB122) in recurrent GBM, with a design that includes preoperative randomization and postoperative combination therapy to assess the impact of dual TIGIT and PD-1 blockade. Another phase I trial (NCT03961971) is exploring the combination of anti-TIM-3 (MBG453) and anti-PD-1 (spartalizumab) with stereotactic radiosurgery, aiming to evaluate safety, the tumor response, and changes in the immune environment.

Although ICIs have transformed the treatment landscape for several cancers, their efficacy in GBM remains limited by a profoundly immunosuppressive TME and challenges in drug delivery. Future strategies focusing on optimized delivery methods, combination regimens, and novel immune targets are essential to overcome these hurdles and improve outcomes in this difficult-to-treat malignancy.

#### CAR-Tcellbased therapy

**Fig. 3:**
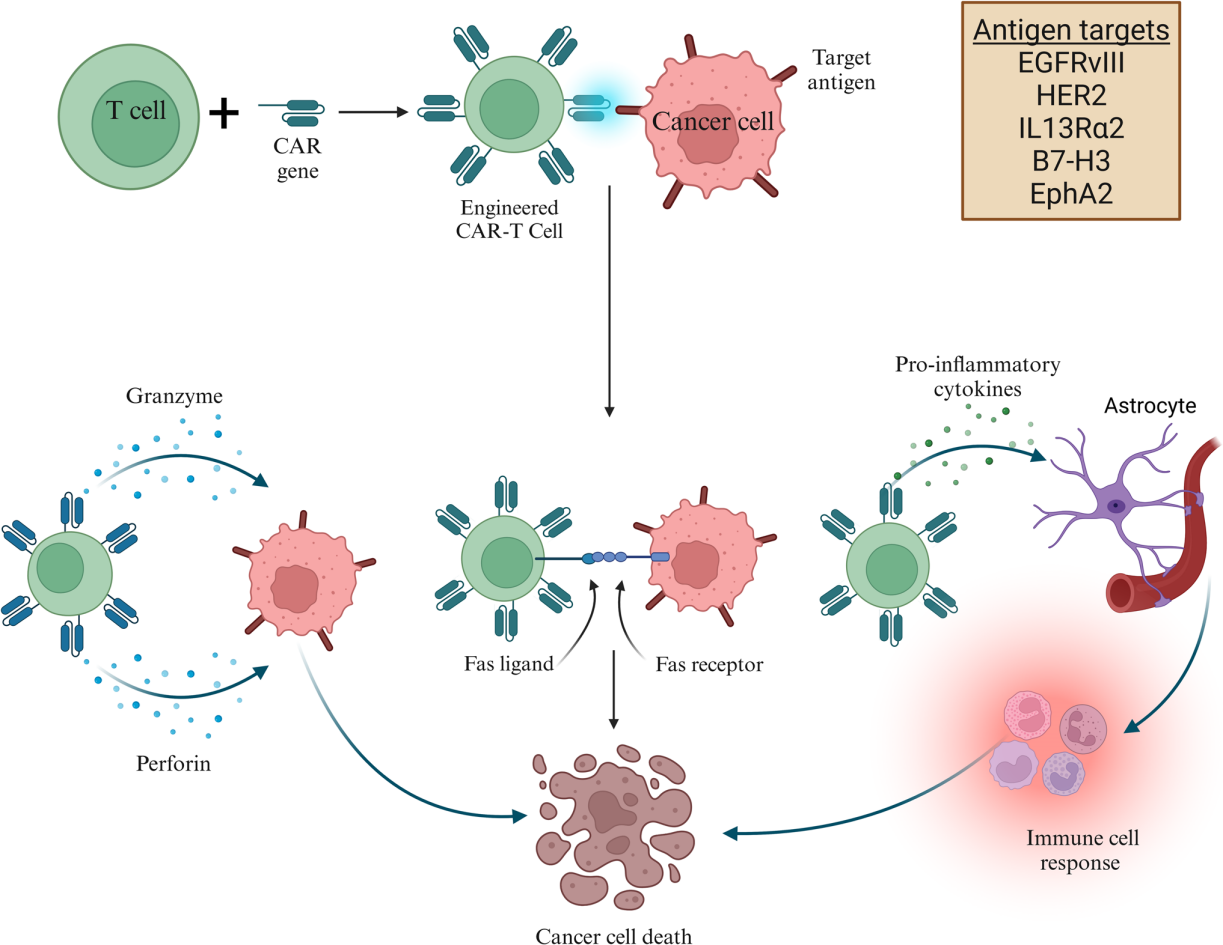
Action of CAR-T cells in GBM: CAR-Tcell therapy in GBM involves modifying patient-derived T cells to express CARs that target tumor antigens (EGFRvIII, HER2, IL13Rα2, B7-H3, EphA2). These engineered cells are expanded and reinfused, where they bind tumor cells and induce death via cytotoxic granule (granzyme, perforin) or Fas ligand–Fas receptor interactions. They also secrete cytokines to recruit immune cells and modulate the microenvironment

Recent advances in GBM immunotherapy have focused on CAR-Tcell strategies to enhance antitumor immunity in this aggressive, immunosuppressive cancer (Fig. 3). Clinical trials (Table S2) have investigated various CAR-Tcell approaches, including IL-13Rα2-targeted CAR-T cells. A phase I trial (NCT02208362) in 65 patients with recurrent high-grade glioma used locally delivered IL-13Rα2 CAR-T cells and achieved a feasible dose of 200 million cells per cycle with dual delivery. The treatment was well tolerated, with only two manageable grade 3 events, and yielded stable disease or better disease in half of the patients, including partial and complete responses. The median overall survival (OS) was 7.7 months, extending to 10.2 months with dual delivery, and increased inflammatory marker levels indicated active immune responses (Brown et al. 2024). A related phase I trial (NCT06815029) at the City of Hope Medical Center is evaluating TGFβR2KO/IL13Rα2 CAR-T cells, which are designed to disable TGFβ signaling, for dose-limiting toxicity, optimal dosing, and early efficacy in recurrent GBM and high-grade IDH-mutant astrocytoma.

EGFRvIII-targeted CAR-Tcell strategies have also been explored. A phase I trial (NCT03726515) in which EGFRvIII CAR-T cells were combined with anti-PD-1 therapy (pembrolizumab) in patients newly diagnosed with EGFRvIII-positive GBM demonstrated safety but limited efficacy, with a median progression-free survival (PFS) of 5.2 months and OS of 11.8 months, while immune analysis revealed increased exhausted and regulatory T cells at relapse (Bagley et al. 2024, p. 20). Another trial (NCT01454596) evaluating EGFRvIII CAR-T cells with adjunct chemotherapy and IL-2 faced severe adverse events, including one death and grade 3 pulmonary toxicity, leading to early termination despite promising preclinical data with CARs derived from mAb139 targeting glioma stem cells. Similarly, a phase I trial (NCT02209376) using CART-EGFRvIII showed on-target activity, with CAR-T cells reaching tumor regions and reducing EGFRvIII levels in some patients, but long-term efficacy was limited by immune resistance and tumor complexity (O'Rourke et al., 2017). A first-in-human trial (NCT05660369) tested CARv3-TEAM-E T cells targeting both EGFRvIII and wild-type EGFR and reported rapid tumor shrinkage in all three treated patients, although the response was short-lived (Choi et al. 2024).

Other CAR-Tcell targets have been explored. A phase I trial (NCT01109095) of HER2-targeted, CMV-specific CAR-T cells in progressive HER2-positive GBM demonstrated safety and clinical benefit in approximately one-third of patients, with some achieving durable responses (Ahmed et al. 2015). A separate phase I trial (NCT06815432) is evaluating GPC3-CAR-T cells enhanced with IL-15 and a safety switch (iCasp9) for recurrent GPC3-positive GBM, in which a single intratumoral dose during surgery with long-term follow-up is planned.

B7-H3-targeted CAR-Tcell therapies have shown promising results. A phase I trial (NCT05241392) using TX103, delivered via an Ommaya reservoir in recurrent GBM, showed no severe toxicity or encouraging efficacy, with 83% of patients surviving at least 12 months and a median OS of 20.3 months, alongside localized CAR-Tcell expansion and increases in inflammatory marker levels in the cerebrospinal fluid (Zhang et al. 2024). A related phase I/II trial at Zhejiang University compared locoregional B7-H3 CAR-T infusions with paused temozolomide chemotherapy to standard treatment, showing early safety and survival benefits. Liquid biopsy studies using CSF and EM-seq have monitored tumor DNA changes in pediatric patients receiving B7-H3 CAR-Tcell therapy, offering real-time insights into treatment response and resistance (Kostecka et al. 2024).

Emerging CAR-T-cell strategies include CLTX-CAR-T cells, which use a scorpion venom-derived peptide that targets MMP2 on GBM cells. A phase I trial (NCT05627323) is evaluating CHM 1101, a CAR-T-cell therapy based on CLTX, in recurrent or progressive GBM through weekly intracranial infusions to assess its safety and feasibility (Badie et al. 2021; Litten et al. 2023). In addition to CAR-T-cell therapy, CAR-NK-cell therapy represents an alternative approach that results in comparable anti-tumor activity. A phase I trial (NCT03383978/CAR2BRAIN) is testing NK-92/5.28.z cells targeting ErbB2 (HER2) in recurrent GBM, investigating safe dosing via intraoperative or reservoir-based delivery, with potential combinations with anti-PD-1 therapy (Li et al. 2018; Burger et al. 2017).

While early-phase trials have demonstrated safety and potential, challenges such as short-lived responses, tumor heterogeneity, and immune resistance highlight the need for optimized dosing, repeat administration, and combination strategies to improve outcomes in this aggressive disease.

#### Cancer vaccines

**Fig. 4:**
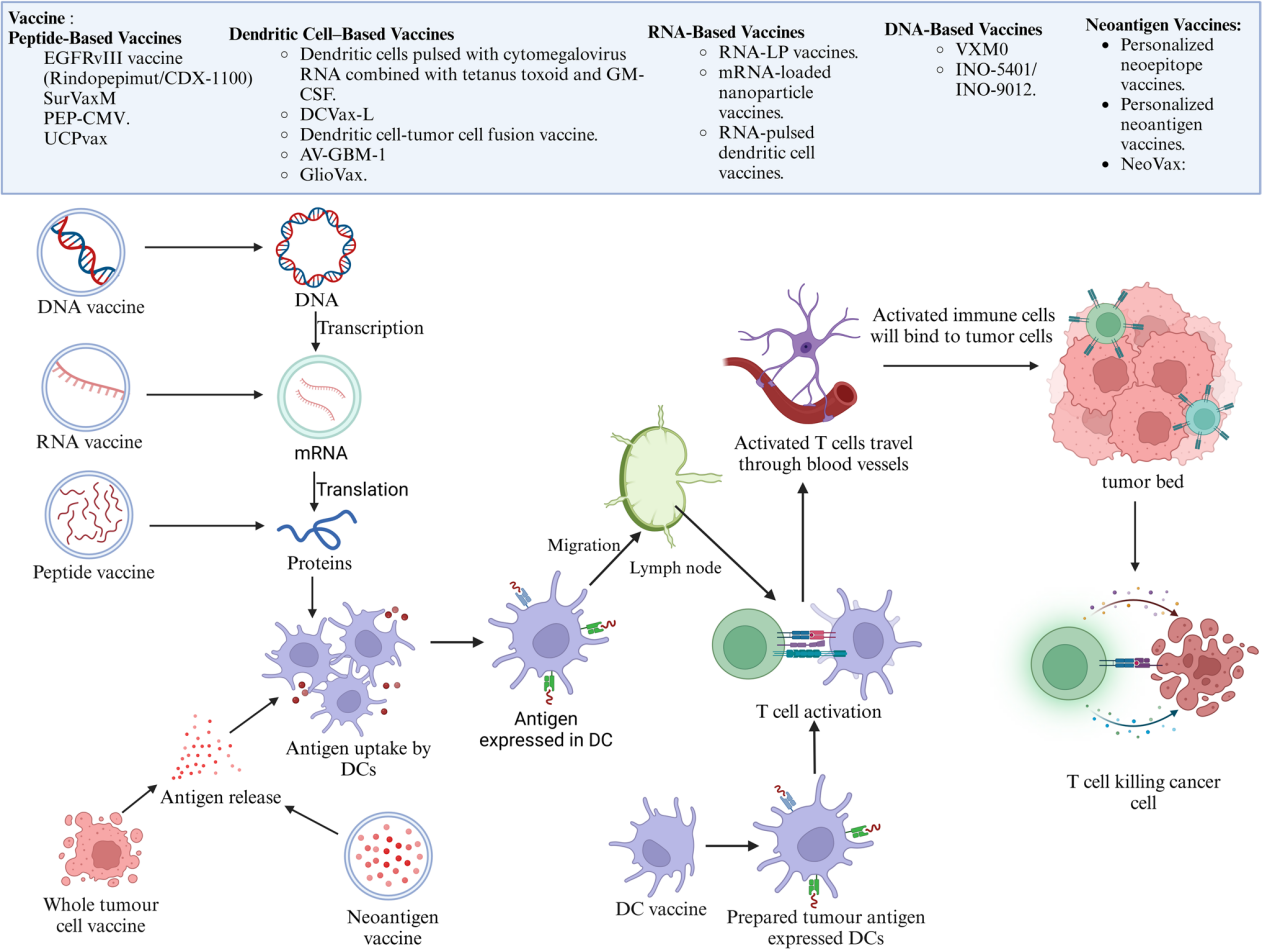
Vaccine-based immunotherapeutic strategies for GBM: The figure highlights vaccine strategies that increase antigen presentation and immune activation in GBM. Peptide vaccines deliver tumor-specific peptides, dendritic cell vaccines use antigen-loaded dendritic cells, RNA and DNA vaccines introduce genetic material for antigen expression, and neoantigen vaccines target tumor-specific mutations. All these strategies promote antigen uptake, T-cell activation, and tumor cell destruction, counteracting immune suppression

GBM vaccines are engineered to target tumor-associated antigens to elicit immune responses that can eradicate malignant cells (Fig. 4). Clinical trials have investigated various vaccine platforms with mixed outcomes (Table S3). For example, a phase II trial of the EGFRvIII vaccine (Rindopepimut/CDX-110) combined with GM-CSF in patients with relapsed EGFRvIII-positive GBM induced antigen-specific responses and improved progression-free and overall survival, especially when it was administered with bevacizumab (Reardon et al. 2015). However, a subsequent phase III trial in newly diagnosed GBM patients receiving standard TMZ was halted because of the absence of significant survival benefits, underscoring the need for innovative approaches (Weller et al. 2017; Binder et al. 2018).

Efforts to improve outcomes continue with novel vaccines and personalized strategies. The SurVaxM vaccine, which was evaluated in a phase II trial with TMZ, achieved a 95% progression-free survival rate at six months in newly diagnosed patients (Ahluwalia et al. 2023). Similarly, personalized approaches using APVAC1 and APVAC2 vaccines demonstrated safety and immunogenicity in phase I trials (Wick et al. 2018). Peptide-based vaccines such as PEP-CMV from a 26-amino acid peptide of the human pp65 cytomegalovirus antigen are under investigation in younger patients, and UCPvax, which targets TERT in unmethylated MGMT GBM patients, elicits strong anti-TERT CD4 + T-cell responses, with a median overall survival of 17.9 months (Thompson et al. 2024; Carpentier et al. 2023).

Dendritic cell-based vaccines have proven safe in pediatric and young adult patients when they are pulsed with cytomegalovirus RNA combined with tetanus toxoid and GM-CSF (Li et al. 2022; Rodriguez et al. 2023). DNA vaccines represent a promising immunotherapeutic strategy in GBM, aimed at eliciting a targeted and sustained immune response against tumor-specific or tumor-associated antigens. The study shows that combining a TRP2-targeted DNA vaccine with dual immune checkpoint blockade (anti-PD-1 and anti-CTLA-4) enhances T cell responses and immune activation in an unresectable GBM model. While survival was not significantly improved, the approach boosted effector T cell activity and offers a promising immunotherapeutic strategy (Bausart et al. 2022). DNA vaccines, such as oral Salmonella typhi-based VXM01, which targets VEGFR-2, have shown favorable safety profiles and detectable T-cell responses, whereas combination therapies with DNA vaccines plus cemiplimab (INO-5401/INO-9012) have demonstrated promising survival outcomes (Londhe and Date 2020; Reardon et al. 2022).

Advanced delivery platforms are also being explored to increase vaccine efficacy. Lipid nanoparticle systems, for example, are being assessed in a phase I trial for RNA‒lipid particle (RNA‒LP) vaccines in recurrent GBM, with a focus on manufacturing feasibility, safety, and maximum tolerated doses (Karimi-Sani et al. 2024). Moreover, a vaccine formed from autologous tumor cells that were genetically engineered to inhibit TGF-β2 induced partial regression of the tumor and increased median survival in a small phase I trial (Fakhrai et al. 2006). A phase III trial of an individualized dendritic cell vaccine (DCVax-L) combined with standard treatment enhanced median overall survival in newly diagnosed and recurrent GBM patients, especially those in the methylated MGMT subgroup (Liau et al. 2023).

Pilot trials further highlight the promise of personalized immunotherapy. A patient-derived tumor vaccine (AFTV) produced from patient tumors produces heterogeneous responses and, in a few instances, leads to prolonged median survival (Ishikawa et al. 2007). Similarly, a dendritic cell–tumor cell fusion vaccine induced a measurable immune response and partial tumor reduction in a small group of patients (Kikuchi et al. 2001). The GlioVax phase II trial revealed that the combination of a personalized dendritic cell vaccine with conventional treatment led to a median survival gain of approximately 32 months in specific subgroups and improved immune responses (Rapp et al. 2018). Studies employing GBM6-AD cell line vaccines, personalized neoantigen vaccines, and AV-GBM-1 dendritic cell vaccines have consistently demonstrated the safety and induction of antitumor immunity, with variable survival results (Rapp et al. 2018; Keskin et al. 2019; Bota et al. 2022).

Current individualized vaccination approaches are promising. NeoVax, engineered by stratification of different tumor regions to counteract GBM heterogeneity, boosted IFNγ-producing T cells and augmented tumor infiltration (Johanns et al. 2024). Furthermore, a phase I trial employing dendritic cells loaded with patient-specific neoantigens administered alongside radiation and chemotherapy demonstrated a favorable safety profile and extended progression-free intervals with robust immune responses (Zhang et al. 2024).

#### Oncolytic virus (OV) therapy

**Fig. 5:**
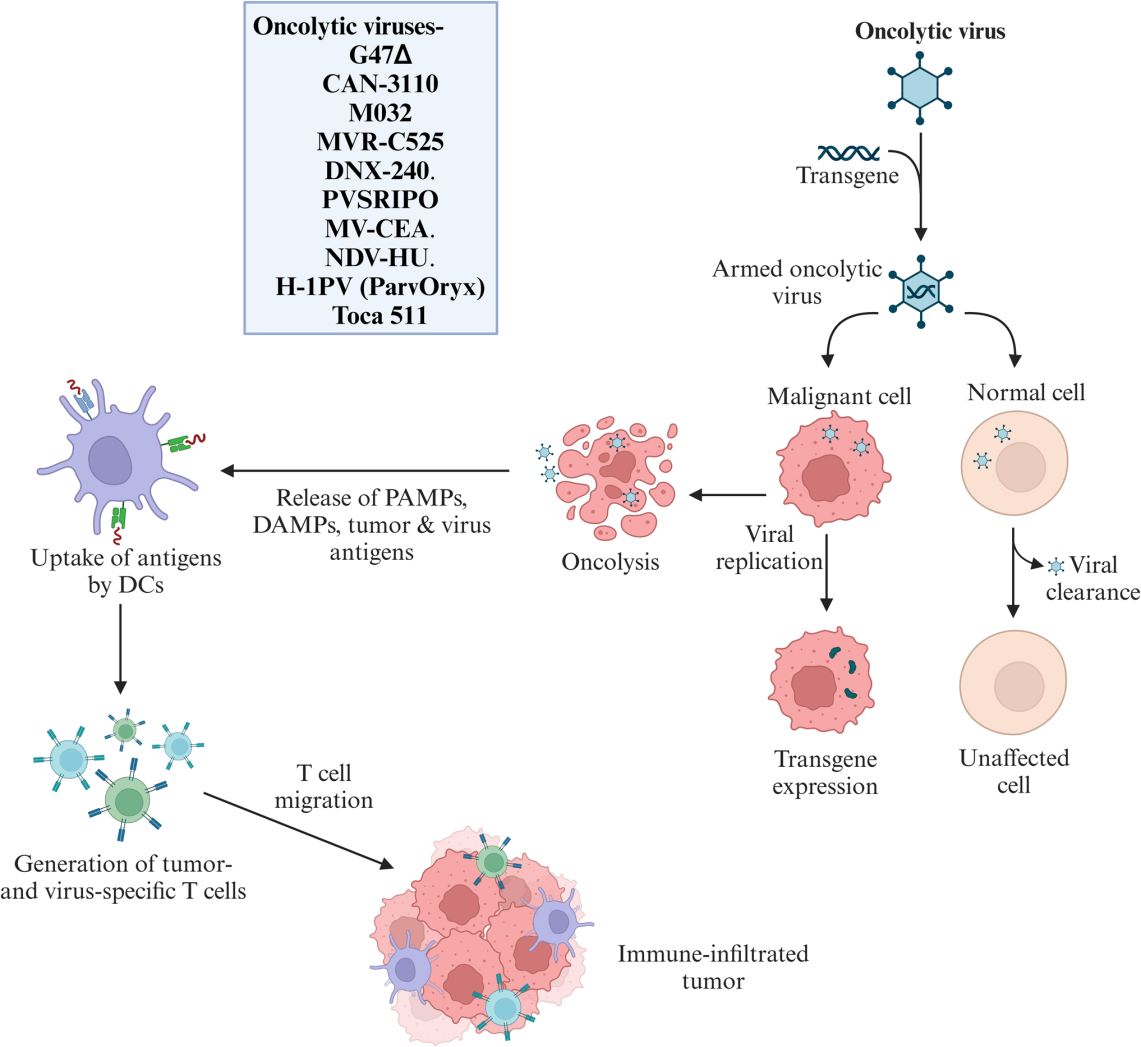
Oncolytic Viruses in GBM Treatment: Schematic illustrating the multistep antitumor action of oncolytic viruses (OVs) in GBM. Engineered OVs selectively infect and replicate in GBM cells, causing oncolysis while sparing normal tissue. This process releases tumor antigens and danger signals, activating dendritic cells to prime T cells, which then expand and migrate to attack both infected and residual tumor cells. This systemic immune response helps overcome immune suppression and may enhance other immunotherapies

OV therapy harnesses genetically engineered viruses to selectively lyse tumor cells and stimulate antitumor immunity; their ability to be genetically modified for improved targeting or therapeutic delivery makes them a promising tool in cancer treatment, especially in combination with immunotherapy (Fig. 5). Several clinical trials (Table S4) have explored different OV strategies for GBM. A phase I/II trial tested G47Δ, a modified herpes virus, in 13 Japanese patients with recurrent GBM. Despite a median survival of 7.3 months, three patients survived more than 3.8 years, with MRI scans confirming tumor cell death and immune activation (Todo et al. 2022a). A phase II trial with 19 patients receiving repeated G47Δ injections reported a one-year survival rate of 84.2% and a median OS of 20.2 months, leading to its approval as Japan’s first OV therapy (Todo et al. 2022b).

Other viral therapies have shown promise. A phase I trial (NCT03152318) evaluated CAN-3110 in recurrent GBM, which demonstrated its safety over four months and enabled molecular analysis (Chiocca et al. 2021). Another trial (NCT05084430) combined M032, an IL-12-expressing herpesvirus, with pembrolizumab to assess dose optimization and efficacy in newly diagnosed GBM patients. MVR-C5252, designed to produce IL-12 and an anti-PD-1 antibody, was tested in a phase I trial (NCT05095441) for safety and viral persistence. The PuMP trial (NCT06126744) evaluated its administration via convection-enhanced delivery to determine the maximum tolerated dose and six-month PFS.

DNX-2401 has demonstrated dual efficacy in phase I trials, with intratumoral injections inducing tumor lysis and immune activation. A study of 37 patients reported durable responses, whereas a phase I/II trial combining DNX-2401 with pembrolizumab in 49 patients reported a median OS of 12.5 months and a one-year survival rate of 52.7% (Lang et al. 2018; Nassiri et al. 2023). PVSRIPO, an attenuated poliovirus, achieved a median survival of 12.5 months in early trials, with follow-up studies exploring its combination with pembrolizumab (Desjardins et al. 2018; Sloan et al. 2021). In pediatric patients, DNX-2401 with RT for DIPG led to a median survival of 17.8 months, offering hope for this difficult subgroup (Pérez-Larraya et al. 2022).

Other viral vectors, including measles virus (MV-CEA) and Newcastle disease virus (NDV-HUJ), have shown safety and positive survival signals, although with variable results (Freeman et al. 2006; Galanis et al. 2024). ParvOryx (H-1PV) and cell-based delivery methods using mesenchymal or neural stem cells have expanded the potential of OV therapy (Fares et al. 2021; Chen et al. 2022). Gene therapy approaches such as Toca 511, which converts Toca FC into 5-FU, showed early promise but failed to demonstrate a survival advantage in a large phase II trial (Robbins et al. 2014; Cloughesy et al. 2020). Other strategies, such as rhenium-186 nanoliposomes, ANG1005 for BBB penetration, and MTX110 for panobinostat delivery, continue to be investigated for GBM treatment (LaFrance et al. 2023; Dmello et al. 2024).

OV therapy holds promise in GBM treatment, but further research is necessary to optimize dosing, delivery methods, and combination strategies to improve patient outcomes. Immunotherapy enhances T cell responses but is limited by GBM’s immunosuppressive environment. DNA vaccines are safe and customizable, inducing specific immune responses, though they often lack strong immunogenicity. Oncolytic virus therapy combines direct tumor lysis with immune activation but faces delivery and antiviral immunity challenges. Each offers unique strengths and limitations in GBM treatment.

### Nanotherapy

**Fig. 6:**
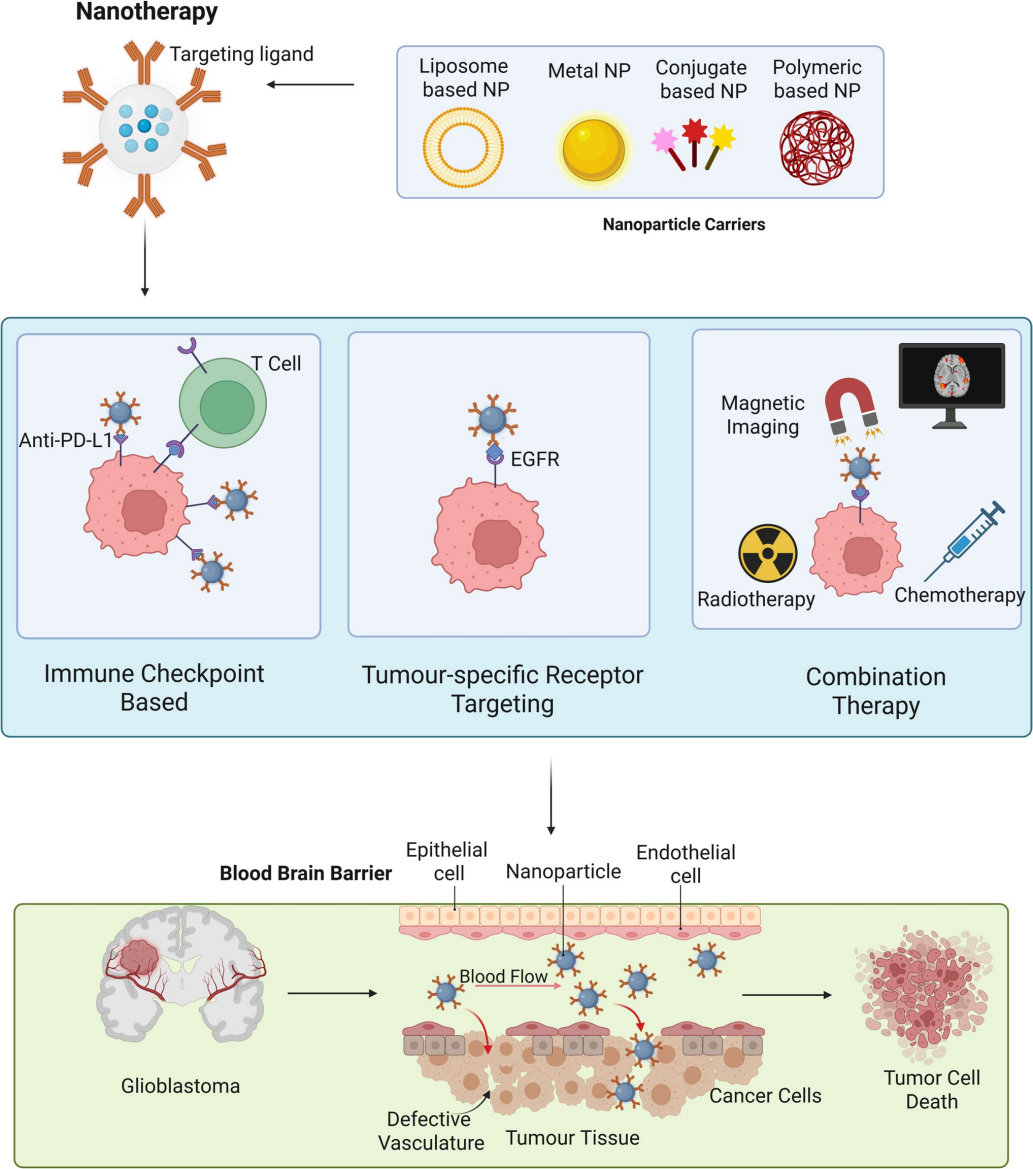
Nanotherapy approach for GBM: The diagram shows that nanoparticle-based delivery systems for GBMs utilize engineered platforms such as liposomes, metals, polymers, and conjugates to transport therapeutics. When functionalized with targeting ligands, these nanoparticles cross the BBB via receptor-mediated transcytosis and accumulate in tumors through the EPR effect and active targeting. Upon internalization, they release their payload, inducing cytotoxicity while minimizing systemic toxicity and enhancing treatment efficacy

Nanoimmunotherapy offers advanced strategies to treat GBM by combining nanotechnology with immunotherapy to overcome major barriers like the BBB and immune suppression (Liu et al. 2022; Nan et al. 2023). Nanotherapy aims to overcome GBM challenges such as poor BBB penetration, treatment resistance, and complex TME (Fig. 6). Nanotherapy is promising for enhancing drug delivery and minimizing side effects, optimizing outcomes especially when used with conventional treatments. Several clinical trials (Table S5) have evaluated nanoparticle-based drug delivery strategies to enhance therapeutic efficacy.

For recurrent GBM, nanotherm therapy (NCT06271421) injects iron oxide nanoparticles postsurgery, inducing hyperthermia under a magnetic field to enhance radiochemotherapy. Early data suggest survival benefits over the Stupp protocol. A phase I trial (NCT03603379) tested anti-EGFR immunoliposomes loaded with doxorubicin in recurrent EGFR-amplified GBM, yielding an 8-month median OS despite limited BBB penetration (Kasenda et al. 2022).

Nanoparticles carrying immune checkpoint inhibitors anti-PD-L1, enable targeted delivery across the BBB, improving local immune responses while minimizing systemic toxicity (Wang et al. 2022). A phase I/II trial (NCT00944801) combined pegylated liposomal doxorubicin (PEG-Dox) with TMZ and RT in 63 newly diagnosed patients. Although well tolerated, with side effects such as hand‒foot syndrome and low blood counts, the 12-month PFS (30.2%) and median OS (17.6 months) did not surpass historical outcomes (Beier et al. 2009). Other trials explored novel formulations. A phase Ib-IIa study (NCT05768919) evaluated liposomal curcumin with RT and TMZ in high-grade gliomas, assessing its safety and early efficacy. AGuIX nanoparticles (NCT04881032) tested with radiochemotherapy improved 6-month PFS and survival, with MRI confirming selective tumor accumulation (Thivat et al. 2023).

Additional strategies include liposomal irinotecan (NCT02022644) in recurrent gliomas, with a median survival of ~ 3.5 months. Intra-arterial chemotherapy (NCT06356883) using carboplatin with liposomal doxorubicin or etoposide aims to bypass the BBB. A phase I trial of V EDV Dox, which targets EGFR, showed safety but limited efficacy, with a median PFS of 1.6 months and an OS of 9.2 months (Whittle et al. 2015).

Gene and RNA therapies have also been investigated. The use of SGT-53 (NCT02340156), a p53-carrying liposome combined with TMZ, has resulted in limited enrollment. An RNA-based therapy (NU-0129, NCT03020017) using spherical nucleic acids to deliver siRNA targeting Bcl2L12 successfully crosses the BBB and reduces target protein levels (Kumthekar et al. 2021).

Radioactive and polymeric formulations include rhenium-186 nanoliposomes (NCT01906385), which deliver localized radiation with a median OS of 12.4 months in recurrent gliomas (LaFrance et al. 2023). ANG1005, a paclitaxel conjugate (NCT01967810), was safe but lacked efficacy (Dmello et al. 2024). MTX110, a water-soluble panobinostat formulation (NCT05324501), was discontinued despite its targeted delivery approach.

Other nanotherapeutics include 2B3-101, a brain-targeted doxorubicin (NCT01386580), which shows promise in the treatment of brain metastases and recurrent gliomas, and nanoliposomal irinotecan with low-dose TMZ (NCT03119064), which is discontinued for inefficacy (Brandsma et al. 2014; Elinzano et al. 2020). In pediatric GBM, Myocet® liposomal doxorubicin (NCT00390299) was used to establish a safe 60 mg/m^2^ dose (Chastagner et al. 2015). These trials highlight the potential of nanotherapies for drug delivery, tumor targeting, and overcoming resistance, although further research is needed to optimize their efficacy and improve survival outcomes. Nanoimmunotherapy enhances and overcomes several limitations of traditional immunotherapy, especially for hard-to-treat cancers like GBM.

## Future directions

The refractory nature of GBM treatment requires a paradigm shift toward more integrative and individualized therapeutic modalities. Future research should address the intricate molecular and genetic heterogeneity of GBM to enhance the stratification of patients and optimize targeted therapy. The innovations in single-cell sequencing, spatial transcriptomics, and predictive modeling with artificial intelligence hold the key to the identification of new biomarkers and mechanisms of resistance and, ultimately, tailored interventions. Furthermore, improving drug delivery across the BBB continues to be a long-term goal. Advances in nanotechnology, such as targeted nanoparticles and drug carriers from extracellular vesicles, may enhance chemotherapy and immunotherapy.

Immunotherapeutic approaches must be maximized to overcome immunosuppressive TME. Complementing tumor vaccines, CAR-Tcell therapy, and oncolytic viruses with immune checkpoint inhibitors may enhance antitumor responses. Thwarting immune escape pathways, including those involving myeloid-derived suppressor cells and regulatory T cells, offers novel therapeutic opportunities. CRISPR gene editing also has the potential for repairing oncogenic mutations and enhancing the immune cell function, which should be pursued further.

New modalities, such as optogenetics, focused ultrasound, and precision radiosurgery, provide minimally invasive tumor control. Computational biology combined with big data has the potential to provide real-time monitoring of disease. Combinatorial strategies for optimal survival and minimal toxicity need to be investigated in future trials.

## Conclusion

GBM is a demanding condition in neuro-oncology because of its invasive growth pattern, molecular heterogeneity, and lack of responsiveness to traditional forms of therapy. Despite growing insights into its pathobiology and the development of innovative therapies, its prognosis remains dismal. The classic approaches of surgery, RT, chemotherapy, and TTF have little survival benefit and thus justify newer treatment modalities.

Recent breakthroughs in immunotherapy, gene-based therapies, and nanotechnology have opened new avenues. However, these methods face a series of challenges in clinical translation, such as tumor heterogeneity, immune evasion, and drug delivery. The future of GBM treatment involves personalized, multimodal approaches that integrate molecular diagnostics, precision medicine, and cutting-edge therapeutic technologies. An in-depth understanding of the biology of GBM and coordination among institutions in translational research and clinical trials will be key to exploring more effective and sustainable treatments. Although GBM is an incurable disease, current developments and multidisciplinary approaches provide promising prospects for improving both survival and quality of life in GBM patients.

## Supplementary Information

Below is the link to the electronic supplementary material.

Supplementary file1 (DOCX 33 KB)

## Data Availability

Not applicable.
