# Supplementary material for: Evolving therapeutic strategies in glioblastoma: traditional approaches and novel interventions
Source: 3 Biotech. 2025 Aug 28;15(9):318. doi: 10.1007/s13205-025-04493-1 (PMC12394108; doi:10.1007/s13205-025-04493-1)
Supplement: Supplementary file 1 — Supplementary file1 (DOCX 33 KB) [file 13205_2025_4493_MOESM1_ESM.docx]

**Table S1:** Checkpoint inhibition clinical trials.

| **Trial ID** | **ICB Therapy** | **Status** | **Phase** | **Key Findings/Outcomes** |
| --- | --- | --- | --- | --- |
| NCT02550249 | Neoadjuvant Nivolumab | Completed | II | Induced immune microenvironment changes, but no clear clinical benefits postsurgery. |
| NCT02337686 | Neoadjuvant Pembrolizumab | Active, not recruiting | II | Limited impact on TME, low T-cell infiltration, predominance of CD68⁺ macrophages. |
| NCT03899857 (PERGOLA) | Pembrolizumab + temozolomide based radiochemotherapy | Active, not recruiting | II | Ongoing trial. |
| NCT02794883 | Tremelimumab + Durvalumab | Completed | II | Evaluating combination vs. monotherapies, endpoints: PFS, OS, immunologic changes. |
| NCT02658981 | Anti-LAG-3 or Urelumab (anti-CD137) ± anti-PD-1 | Completed | I | Prolonged survival in a subset of patients. |
| NCT02335918 | Anti-CD27 + Nivolumab | Completed | I/II | combination of varlilumab and nivolumab was well tolerated but overall efficacy was not greater than expected for nivolumab |
| NCT04656535 | Domvanalimab (AB154) + Zimberelimab (AB122) | Active, not recruiting | 0/I | Testing safety and immune effects of TIGIT + PD-1 blockade. |
| NCT03961971 | Anti-TIM-3 (MBG453) + Anti-PD-1 (spartalizumab) + Stereotactic Radiosurgery (SRS) | Active, not recruiting | I | Testing safety and efficacy of combination therapy. |
| NCT02017717 (CheckMate 143) | Nivolumab vs Bevacizumab | Completed | III | Nivolumab did not improve overall survival. |
| NCT02617589 (CheckMate 498) | Nivolumab + Radiotherapy | Completed | III | Failed to meet primary endpoints for overall survival. |
| NCT02638902 (CheckMate 548) | Nivolumab + Radiotherapy + temozolomide | Completed | III | Failed to meet primary endpoints for overall survival. |
| NRG BN007 | Nivolumab + Ipilimumab | Closed to Accrual & Treatment | II/III | Failed to meet primary endpoints for overall survival. |

**Table S2:** CAR-T-based therapies and their clinical trials.

| **Trial ID** | **CAR Target/Therapy** | **Status** | **Phase** | **Key Findings/Outcomes** |
| --- | --- | --- | --- | --- |
| NCT02208362 | IL-13Rα2 CAR T cells | Active, not recruiting | I | Safe, stable disease or better in 50% of patients, median OS: 7.7 months (10.2 months with dual delivery). Increased inflammatory markers. |
| NCT06815029 | TGFβR2KO/IL13Rα2 CAR T cells | Not yet recruiting | I | Evaluating safety, optimal dosing, and initial effectiveness. |
| NCT03726515 | EGFRvIII CAR T cells + Pembrolizumab | Completed | I | Safe, median PFS: 5.2 months, median OS: 11.8 months, increased exhausted/regulatory T cells at relapse. |
| NCT01454596 | EGFRvIII CAR T cells + Chemotherapy + IL-2 | Completed | I/II | Terminated early due to severe adverse events. |
| NCT02209376 | CART-EGFRvIII | Terminated | I | On-target activity, CAR T cells reached tumor, reduced EGFRvIII in some, limited long-term efficacy due to immune resistance. |
| NCT01109095 | HER2 CAR T cells (CMV-specific) | Completed | I | Safe, clinical benefit in ~33% of patients, durable responses. |
| NCT05241392 | B7-H3 CAR T cells (TX103) | Active, not recruiting | I | Safe, 83% 12-month survival, median survival: 20.3 months, increased CAR T cells and inflammatory markers in CSF. |
| NCT05241392 | B7-H3 CAR T cells | Active, not recruiting | I | Comparing locoregional infusions with paused TMZ to standard treatment, early signs of safety and potential survival benefit. |
| NCT05835687 | B7-H3 CAR T cells (CSF liquid biopsies, EM-seq) | Recruiting | I | Monitoring tumor DNA changes in CSF, real-time insights into treatment response and resistance. |
| NCT04214392 | CLTX-CAR T cells (MMP2) | Recruiting | I | Assessing safety, optimal dosing, and early efficacy. |
| NCT05627323 | CHM 1101 (CLTX-based CAR T cells) | Active, not recruiting | Ib | Safety, feasibility, and early efficacy with weekly intracranial infusions. |
| NCT05660369 | CARv3-TEAM-E T cells (EGFRvIII + wild-type EGFR) | Recruiting | I | Rapid tumor shrinkage, short-lived responses in most cases. |
| NCT06815432 | GPC3-CAR T cells (+IL15, iCasp9 safety switch) | Not yet recruiting | I | Intratumoral dose, long-term follow-up planned. |
| NCT03383978/CAR2BRAIN | ErbB2 (HER2) CAR-NK cells (NK-92/5.28.z) | Active, not recruiting | I | Determining safe dosing, exploring combinations with anti-PD-1. |

**Table S3:** Clinical trials on vaccines.

| **Trial ID** | **Vaccine/Therapy** | **Status** | **Phase** | **Key Findings/Outcomes** |
| --- | --- | --- | --- | --- |
| NCT01498328 | EGFRvIII (Rindopepimut/CDX-110) + GM-CSF | Completed | II | Encouraging PFS/OS, improved survival with bevacizumab. |
| NCT01480479 | Rindopepimut/GM-CSF | Completed | III | Discontinued, failed to improve survival. |
| NCT02455557 | SurVaxM + temozolomide | Active, not recruiting | II | 95% 6-month PFS, immune responses against survivin. |
| NCT02149225 | APVAC1/APVAC2 (personalized) | Completed | I | Safe, immunogenic vaccines. |
| NCT02287428 | Personalized neoepitope vaccines | Recruiting | I | Feasible, safe, robust immune responses. Dexamethasone suppressed responses. |
| NCT03299309 (PRiME) | PEP-CMV (peptide) | Active, not recruiting | I | Actively recruiting. |
| NCT04280848 | UCPvax (TERT) | Completed | II | High immunogenicity, median OS: 17.9 months. |
| NCT06389591 | RNA-LP vaccines | Recruiting | I | Assessing feasibility, safety, and MTD. |
| NCT03615404 | Dendritic cells (CMV RNA) | Completed | I | Safe, common side effects: lymphopenia, headaches. |
| NCT02718443 | VXM01 (DNA vaccine) | Completed | I | Safe, VEGFR-2-specific T-cell responses, potential benefit with anti-PD-L1. |
| NCT04015700 | DNA vaccine (IDH1/IDH2 mutant) | Active, not recruiting | I | Investigating DNA vaccine, INO-9012, and GNOS-PV01. |
| NCT03491683 | INO-5401 + INO-9012 + Cemiplimab | Active, not recruiting | I/II | Safe, median survival: 17.9 months (unmethylated MGMT), 32.5 months (methylated MGMT). |
| NCT04573140 | RNA-loaded nanoparticle vaccines | Recruiting | N/A | Delivering tumor-specific mRNA to immune cells. |
| NCT00045968 | DCVax-L (dendritic cell) | Unknown status | III | Extended survival in both newly diagnosed and recurrent GBM. |
| NCT03395587 (GlioVax) | Dendritic cell vaccine | Active, not recruiting | II | Safe, longer median survival, delayed tumor progression. |
| NCT02549833 | GBM6-AD + poly-ICLC | Completed | Pilo/I | Safe, strong immune responses in presurgery group. |
| NCT02287428 | Personalized neoantigen vaccine | Recruiting | I | Strong T-cell responses, median survival: 16.8 months. |
| NCT03400917 | AV-GBM-1 (dendritic cell) | Unknown status | II | Safe, extended median time without tumor progression, but no significant improvement in OS. |
| NCT03422094 | NeoVax (personalized) | Terminated | N/A | Increased immune cells, T cells targeting tumor. |
| NCT04968366 | Dendritic cell vaccine (neoantigens) | Terminated | I | Safe, promising early results, delayed cancer progression. |

**Table S4:** Clinical trials of oncolytic viruses.

| **Trial ID** | **Virus/Therapy** | **Status** | **Phase** | **Key Findings/Outcomes** |
| --- | --- | --- | --- | --- |
| UMIN000015995 | G47Δ | Completed | I/II | Median OS: 7.3 months (Phase I/II), 20.2 months (Phase II). Safe, some long-term survivors. Approved in Japan. |
| NCT03152318 | CAN-3110 | Recruiting | I | Safe, detailed molecular analysis of tumor changes. Higher-dose group planned. |
| NCT05084430 | M032 + Pembrolizumab | Recruiting | I/II | Phase I: Determining safe dose. Phase II: Evaluating efficacy in newly diagnosed patients. |
| NCT05095441 | MVR-C5252 (C5252) | Not yet recruiting | I | Testing escalating doses, assessing safety and early efficacy. |
| NCT06126744 | MVR-C5252 (PuMP Trial) | Recruiting | I | Testing MVR-C5252 with convection-enhanced delivery (CED). Determining maximum tolerated dose (MTD). |
| NCT00805376 | DNX-2401 | Completed | I | Safe, 20% of patients survived >3 years. Tumor shrinkage and immune response observed. |
| NCT03896568 | MSC-DNX-2401 | Recruiting | I | Precise delivery using MRI/CT fusion. Early results show accurate targeting. |
| NCT01582516 | Delta-24-RGD | Completed | I/II | Testing safety and effectiveness with convection-enhanced delivery. |
| NCT02798406 | DNX-2401 + Pembrolizumab | Completed | I/II | Safe, promising survival outcomes (52.7% 1-year survival). |
| NCT03178032 | DNX-2401 + Radiotherapy | Completed | I/II | Median survival: 17.8 months, promising survival in children. |
| NCT05914935 | YSCH-01 | Recruiting | I | Testing safety and efficacy of YSCH-01 with Ommaya reservoir. |
| NCT00390299 | MV-CEA | Completed | I | Safe, median survival: 11.6 months, immune activity observed. |
| NCT02444546 | Sargramostim + Pelareorep | Completed | I | Tolerable, but no disease control. |
| NCT01491893 | PVSRIPO | Completed | I | Safe, median survival: 12.5 months, durable survival benefits. |
| NCT02986178 | PVSRIPO | Active, not recruiting | II | Evaluating safety, tumor response, and survival. |
| NCT04479241 | PVSRIPO + Pembrolizumab | Completed | II | Testing combination therapy for improved survival. |
| NCT01174537 | Newcastle Disease Virus (NDV) | Withdrawn | I/11 | Trial completed, but outcome details not provided. |
| NCT01301430 | ParvOryx (H-1PV) | Completed | I/II | Safe, some tumor responses, potential anticancer activity. |
| NCT06504381 | DB107-RRV + DB107-FC | Recruiting | I/II | Testing combination with standard therapies. |
| NCT01156584, NCT01470794, NCT01985256 | Toca 511/FC | Completed | I | Safe, some tumor shrinkage, promising early survival. |
| NCT02414165 | Toca 511/FC | Terminated | II/III | No survival benefit compared to standard care. |
| NCT03072134 | NSC-CRAd-S-pk7 (neural stem cells) | Completed | I | Safe, promising survival, increased immune cell activity. |

**Table S5:** Clinical trials on nanotherapy.

| **Trial ID** | **Nanoparticle/Therapy** | **Status** | **Phase** | **Key Findings/Outcomes** |
| --- | --- | --- | --- | --- |
| NCT00944801 | PEG-Dox + temozolomide + Radiotherapy | Completed | I/II | 12-month PFS: 30.2%, Median OS: 17.6 months. Well-tolerated but did not surpass historical outcomes. |
| NCT06271421 | NanoTherm (iron oxide nanoparticles) | Recruiting | N/A (Early data) | Potential to extend survival compared to the standard Stupp protocol. |
| NCT03603379 | Anti-EGFR immunoliposomes (doxorubicin) | Completed | I | Median OS: 8 months. High doxorubicin concentration in disrupted BBB regions. |
| NCT05768919 | Liposomal curcumin + temozolomide + Radiotherapy | Recruiting | I/II | Evaluating safety, dosing, and early efficacy. |
| NCT04881032 | AGuIX nanoparticles + Radiochemotherapy | Active, not recruiting | I/II | Improved 6-month PFS, better neurological outcomes, selective tumor accumulation. |
| NCT02022644 | Liposomal irinotecan (nal-IRI) | Completed | I | Limited clinical efficacy, Median survival ~3.5 months. |
| NCT06356883 | Carboplatin + liposomal doxorubicin/etoposide phosphate (intraarterial) | Recruiting | II | Aim to bypass BBB, enhance local drug concentration. |
| NCT01967810 | ANG1005 (paclitaxel conjugate) | Completed | II | Acceptable safety, but failed to show significant efficacy, development halted. |
| NCT05324501 | MTX110 (panobinostat) | Terminated | I | Terminated early for strategic reasons. |
| NCT01386580 | 2B3-101 (brain-targeted doxorubicin) | Completed | I/II | Promising tumor responses. |
| NCT03119064 | Nanoliposomal irinotecan + low-dose temozolomide | Terminated | I/II | Halted due to lack of efficacy. |
| NCT00390299 | Myocet (nonpegylated liposomal doxorubicin) | Completed | I | Determined safe dose: 60 mg/m² every 3 weeks. |
| NCT01906385 | Rhenium-186 nanoliposomes (186RNL) | Recruiting | I/II | Median survival: 12.4 months. High localized radiation doses. |
| NCT02340156 | SGT-53 (p53 gene liposome) + temozolomide | Terminated | II | Minimal early enrollment. |
| NCT03020017 | NU-0129 (Bcl2L12 siRNA SNAs) | Completed | I | Crossed BBB, reduced target protein, promising precision treatment. |
